# Nonoperative management of appendiceal phlegmon or abscess in children less than 3 years of age

**DOI:** 10.1186/s13017-018-0170-9

**Published:** 2018-03-02

**Authors:** Hailan Zhang, Yuzuo Bai, Weilin Wang

**Affiliations:** Department of Pediatric Surgery, Shengjing Hospital of China Medical University, No. 36 SanHao St., Heping District, Shenyang, 110004 China

**Keywords:** Appendiceal phlegmon, Appendiceal abscess, Nonoperative treatment, Children, Appendicolith

## Abstract

**Background:**

In children less than 3 years of age, there is little experience in the nonoperative management of appendiceal phlegmon or abscess (APA), especially in APA with an appendicolith. The purposes of this study were to evaluate the effects of an appendicolith and the success rate of nonoperative management for APA in these young children.

**Methods:**

Children younger than 3 years of age with APA who underwent attempted initial nonoperative treatment between January 2008 and December 2016 were reviewed. Based on the presence or absence of an appendicolith on admission ultrasonography examination or computed tomography scan, children were divided into two groups: appendicolith group and no appendicolith group.

**Results:**

There were 50 children who met the study criteria. Among 50 children, three children failed to respond to nonoperative treatment because of aggravated intestinal obstruction or recurrent appendicitis within 30 days of admission. The overall success rate for nonoperative management of APA was 94% (47/50) in children younger than 3 years old. The rate of diarrhea and CRP levels were higher in the appendicolith group than that of the no appendicolith group (*P* < 0.05). However, the success rate and the hospital length of stay for nonoperative treatment in the appendicolith group and the no appendicolith group were similar without statistical significance.

**Conclusion:**

APA with or without an appendicolith can have nonoperative management without immediate appendectomy in children less than 3 years old.

## Background

Acute appendicitis is the most common surgical emergency in children [1]. In young children less than 3 years of age, however, the incidence of appendicitis is low (approximately 2.3% of all children with acute appendicitis); thus, appendicitis in this age group is easily missed by pediatricians [2]. Furthermore, in young children, the clinical symptoms and physical signs of appendicitis are atypical and frequently overlap with other common gastrointestinal diseases. Therefore, delayed diagnosis and a higher perforation rate occur more often [3–5]. The appendiceal phlegmon or abscess (APA) at presentation occurs in about 33 to 50% in this young age group [6, 7]. The optimal management of APA is controversial, especially in APA with an appendicolith. The debates predominantly focus on the effects of an appendicolith and the success rate of nonoperative treatment. Some surgeons preferred immediate operation for APA [8], whereas some researches supported nonsurgical management with antibiotics because appendectomy could be technically difficult and complication rates rose [9–12]. The presence of an appendicolith might predict failure of nonoperative treatment of APA, and immediate appendectomy may be a better choice [13]. Some studies found no correlation between clinical outcomes and the presence of an appendicolith [14]. Current studies mainly focused on adults and older children; however, little experience exists with the nonoperative treatment of APA during the first 3 years of life. The purposes of this retrospective study were to evaluate the appendicolith effects and the success rate of nonoperative management for APA in children under 3 years old.

## Methods

### Patients

The medical records of all pediatric patients (< 3 years of age) with APA who underwent attempted initial nonoperative treatment between January 2008 and December 2016 were reviewed. There were no signs of generalized peritonitis or apparent intestinal obstruction among the patients. The data collected included the patient’s characteristics, duration of symptoms, common symptoms (e.g., pain, fever, vomiting, and diarrhea), physical examinations (e.g., mass, tenderness, rebound, and rigidity), white blood cell (WBC) counts, C-reactive protein (CRP) levels, antibiotics administered, length of stay (LOS), ultrasonography (US) findings, and computed tomography (CT) scan findings.

Treatment failure with nonoperative management was defined as unsuccessful in case of an appendectomy during the initial hospitalization or a subsequent readmission within 30 days of admission. Periappendiceal abscesses were not generally drained unless the condition of children did not improve or abscesses gradually increased. The nonsurgically treated children were given intravenous, broad-spectrum antibiotics such as cefoperazone and sulbactam sodium and ceftriaxone and tazobactam sodium. The therapy was continued for at least 5 days. When the patients improved, US or CT was repeated. If regression of appendiceal inflammation was noted on US or CT and the children remained afebrile, physical signs improved, and the WBC counts and CRP levels decreased; the children were discharged home with oral broad-spectrum antibiotics such as cefdinir and cefaclor.

### Statistical analysis

Data were presented as mean ± standard deviation. Statistical evaluation of data was performed by independent-samples *t* test and chi-square test (Fisher’s exact test was used when *T* < 5) depending on data (SPSS 17.0, SPSS, Chicago, IL). A *P* value of < 0.05 was considered as significant.

## Results

Fifty children less than 3 years old underwent attempted initial nonoperative management during the study period. There were 29 boys and 21 girls, with an average age of 24.60 ± 8.49 months (range 5–35 months). The mean duration of symptoms was 8.58 ± 5.49 days. The most common symptoms were fever in 48 patients (96%), abdominal pain in 42 (84%), vomiting in 25 (50%), and diarrhea in 24 (48%). Tenderness was found in all children; 30 of them (60%) also presented localized peritonitis. The WBC counts and CRP levels were 19.20 ± 6.37 × 10^9^/L and 100.27 ± 74.73 mg/L, respectively. Mean inflammatory areas of APA were 20.08 ± 14.77 cm^2^. Two children underwent US-guided percutaneous drainage. One child with an appendicolith underwent early appendectomy because of aggravated intestinal obstruction, while two children without an appendicolith required subsequent readmission because of recurrent pain within 30 days of admission. They responded to antibiotics with complete resolution clinically and were discharged 5 days later. The hospital LOS was 13.24 ± 5.41 days and the overall success rate for nonoperative management of APA was 94% (47/50).

Among 50 children, 46 (92%) were performed with admission US; 45 (90%) were examined with CT scans. Based on the presence or absence of an appendicolith on admission US or CT imaging, the patients were categorized into two groups: appendicolith group [*n* = 27] and no appendicolith group [*n* = 23]. A summary of patient characteristics is displayed in Table 1. No difference existed between the appendicolith group and the no appendicolith group when comparing age, duration of symptoms, pain, fever, vomiting, localized peritonitis, WBC, and mean inflammatory areas. The rate of diarrhea (63.0 vs. 30.4%) and CRP levels (126.83 ± 83.46 vs. 69.10 ± 48.21) were higher in the appendicolith group than the no appendicolith group (*P* < .05). The overall success rates (96.3 vs. 91.3%) and LOS (14.26 ± 5.55 vs.12.04 ± 5.09) for nonoperative treatment in the appendicolith group and the no appendicolith group were similar without statistical significance.

**Table 1:** Clinical characteristics between the appendicolith group and no appendicolith group

	Appendicolith (*n* = 27)	No appendicolith (n = 23)	*P* value
Sex (male:female)	15:12	14:9	0.704
Age (months)	25.89 ± 7.47	23.09 ± 9.49	0.249
Duration of symptoms	8.04 ± 4.17	9.22 ± 6.77	0.472
Pain	23 (85.2%)	19 (82.6%)	0.804
Fever	26 (96.3%)	22 (95.7%)	1.000
Vomiting	12 (44.4%)	13 (56.5%)	0.395
Diarrhea	17 (63.0%)	7 (30.4%)	0.022*
Localized peritonitis	19 (70.4%)	11 (47.8%)	0.105
WBC	20.69 ± 6.79	14.44 ± 5.46	0.072
CRP	126.83 ± 83.46	69.10 ± 48.21	0.004*
Inflammatory area (cm^2^)	21.58 ± 15.15	18.32 ± 14.45	0.442
LOS	14.26 ± 5.55	12.04 ± 5.09	0.151
Overall success	26 (96.3%)	21 (91.3%)	0.588

*P < 0.05

## Discussion

The peak incidence of acute appendicitis occurs in the second decade of life, while it is uncommon to face appendicitis in children younger than 3 years of age. The incidence is approximately 2.3% of all children with acute appendicitis in this young age group [2]. Inability of a young child to communicate to the parents or clinicians, atypical presentation, and other associated illness may delay the diagnosis. So, perforated appendices could be already present in 60–100% when the diagnosis is performed in young children [3, 15, 16]. Some surgeons preferred immediate operations even if intra- and post-operative complications were higher because the anatomic immaturity and an inadequate omental barrier of young children might lead to diffusion of appendiceal inflammation. However, some studies had reported that the ability to localize intraperitoneal inflammatory processes was well-developed. An appendix mass is discovered at the time of presentation in about 33 to 50% of young children under 3 years of age [6, 7].

Although some reviews report antibiotic therapy is safe and effective [17–20], first-line treatment of acute appendicitis remains to be appendectomy, especially laparoscopic appendectomy [21–23]. The best treatment strategy for APA is very controversial. Immediate appendectomy may be technically demanding because of the distorted anatomy and the difficulties to close the appendiceal stump because of the inflamed tissues. It is rare but it exists that the exploration has to end up in an ileocaecal resection or a right-sided hemicolectomy due to the technical problems or a suspicion of malignancy because of the distorted tissues [8, 24, 25]. So, nonoperative management is suggested because of a good and extensively documented success rate [10, 26]. The disadvantages of nonoperative management are that the actual pathology remains unclear and that appendicitis can “recur” after successful nonsurgical treatment. Many researches indicated the risks of recurrence and undetected serious diseases were very low and supported the nonoperative treatment [10, 26–30]. In 2015, the World Society of Emergency Surgery Jerusalem guidelines also suggested that nonoperative management was a reasonable first-line treatment for APA [12]. Weber and Di Saverio advocated for a clinical approach that is tailored to the individual patient’s circumstances and reflective of the situational realities of each patients [31]. However, these researches mainly focus on adults and older children. Little experience exists with the nonoperative treatment of APA in children less than 3 years old.

The success rate for nonoperative treatment of APA was 94% during the first 3 years of life in our review. This is similar to previous researches that the success rate ranged from 84 to 98% in adults and children [10, 26, 32, 33]. The post-operative complications of immediate appendectomy were obvious higher, and laparoscopic appendectomy occurred significantly less in children younger than 3 years [2, 34–36]. These indicated the nonoperative management of APA is superior to immediate appendectomy in children younger than 3 years of age.

Three children with APA failed to respond to nonoperative treatment. Among them, two patients returned to the hospital and required intravenous antibiotics and an admission of 5 days because of recurrent pain. One child deteriorated during antibiotic therapy and required early appendectomy. The rate of early appendectomy was only 2% during the nonoperative treatment in young children under 3 years old. Our high success rate of nonoperative management might be connected with our nonoperative strategy. Generalized peritonitis or apparent intestinal obstruction was excluded from the nonsurgical treatment of APA. Under these clinical conditions, it was difficult to succeed according to our experiences to treat APA in older children.

An appendicolith, or fecalith, is composed of inspissated fecal material, mucus with entrapped calcium phosphate, and inorganic salts. The appendicolith has long been implicated as an important cause of APA [37]. When an appendicolith was present in APA, it was believed to predict failure of nonsurgical therapy and immediate appendectomy was suggested [13]. However, some researches indicated that APA with an appendicolith can be managed nonoperatively and immediate appendectomy is not necessary in older children [14, 26]. To our knowledge, nonoperative management of APA with an appendicolith has not been systematically reported in children younger than 3 years old.

With respect to the influence of appendicoliths on nonoperative treatment of APA, the rate of diarrhea and CRP levels were higher in the appendicolith group compared to the no appendicolith group; however, these have no influence on the nonoperative treatment outcomes, including the hospital LOS and overall success rate. The nonoperative success rate of the appendicolith group was 96.3%. Hence, APA with an appendicolith can be treated nonoperatively and the presence of an appendicolith does not affect the therapeutic effect in young children less than 3 years of age.

It is allowed to diagnose children appendicitis by CT scan in our country and Simanovsky N et al. had reported non-diagnostic or equivocal US in the evaluation of acute abdominal pain in children younger than 10 years old is probably sufficient to justify the additional CT radiation burden [38]. CT scanning had been done in most young children because the ultrasonic diagnosis was uncertain, little experience existed with the nonoperative treatment of APA, and the effect of an appendicolith on clinical outcomes was unclear. The diagnostic performance of US depends on the technique of the examiners, and it is not always easy to detect the presence or absence of an appendicolith in APA. Now, the use of CT scan had been obviously decreased in our hospital because of higher success rate for nonoperative treatment and no correlation between clinical outcomes and the presence of appendicolith if APA could be diagnosed by US.

This review has some limitations. It was a single-center research. The number of patients was small because of the lower morbidity and the higher pre-operative misdiagnosis. Another limitation was that the data were retrospectively collected. These might have resulted in some degree of bias. Additional prospective trials with a larger number of subjects are needed to validate our conclusions about the optimal management of APA in young children.

## Conclusions

The presence of an appendicolith does not affect the nonoperative therapeutic effect of APA in young children. In the absence of generalized peritonitis or apparent intestinal obstruction, APA can be managed nonoperatively without immediate appendectomy in children less than 3 years old.
